# A Clarion Call for Neuroinflammatory Assays in Brain Stimulation for Chronic Pain

**DOI:** 10.3390/brainsci12030364

**Published:** 2022-03-09

**Authors:** Thomas Kinfe, Yining Zhao, Barbara Viviani

**Affiliations:** 1 Division of Functional Neurosurgery and Stereotaxy, Friedrich-Alexander University (FAU) Erlangen-Nürnberg, 91054 Erlangen, Germany; yining.zhao@uk-erlangen.de; 2 Department of Neurosurgery, Friedrich-Alexander University (FAU) Erlangen-Nürnberg, 91054 Erlangen, Germany; 3 Department of Pharmacological and Biomedical Sciences, Università degli Studi di Milano, 20133 Milan, Italy; barbara.viviani@unimi.it

**Keywords:** brain stimulation, chronic pain disorders, neuroinflammation, cytokine, adipokine, brain–immune communication

Chronic pain is characterized by an impaired functional state (pain, mood, sleep, cognition, and metabolism) affecting different brain networks relevant for pain perception and neural pain processing. Regardless of the origin, chronic pain poses a therapeutic challenge facing refractority and the side effects of pharmacotherapies in a considerable subset of chronic pain sufferers, leading to the urgent need of adjunctive noninvasive as well as invasive brain stimulation strategies [1,2,3]. Based upon a therapy-resistant chronic pain state, noninvasive ((NIBS: transcranial magnetic stimulation (TMS), transcranial direct current stimulation (tDCS), and transcranial alternating current stimulation (tACS)) and invasive brain modulation approaches (deep brain stimulation (DBS) and epidural brain stimulation (MCS)) have yielded a meaningful form of pain relief, depending on the applied methodology. However, as very little robust and high quality comparative data (noninvasive versus invasive brain stimulation) are available on this subject, evidence-derived selection in favor or against one therapeutic option appears to be difficult. Furthermore, comparative, sham-controlled trials may be biased, when comparing noninvasive versus invasive brain stimulation in one study cohort. Finally, a decision-making clinical framework may encompass a staged approach considering NIBS therapies (TMS, tDCS, and tACS) to be applied first, and, secondly, if a limited response is present, invasive treatment options (DBS and MCS) may represent further treatment options. Nevertheless, some chronic pain individuals may prefer a reversible NIBS approach (TMS, tDCS, and tACS) over a reversible invasive approach (DBS and MCS). Notably, NIBS and DBS do not exclude each other in that way, so that TMS may support DBS by developing a neural network-based interventional strategy prior to DBS [4,5,6].

Experimental as well as in-human studies indicate that chronic pain stems from an upregulated central and peripheral neuroinflammatory axis, which is mediated by a vast array of signaling molecules and cellular components relevant for chronic pain pathophysiology [7,8]. This cytokinic microenvironment derives either from resident macrophages in the brain (microglia) and in the peripheral nervous system (satellite glial cells), together with the contribution of other circulating immune cells (e.g., neutrophils, basophils, and mast cells).

Pain-related inflammation may be consequent to tissue damage or to general conditions leading to a “low grade persistent inflammation.”, as observed in obesity [7]. In addition to controlling the activity of non-neuronal cells, cytokines potently modulate neuronal functions due to the expression of specific receptors [8]. Thus, the peripheral wave of cytokines initiates a complex crosstalk by tuning thermal, mechanical, or chemical stimuli at nociceptors, which, in turn, stimulates the excitation of second-order neurons in the spinal cord. Additionally, the functional connection between neurons along the pain pathway (DRG, second-order neurons in the dorsal horn of the spinal cord and neurons in the supra-spinal areas) and the surrounding glial cells is crucial in propagating the peripheral cytokine wave up to the cerebrum areas. As a result of this activity, cytokines engage highly plastic circuits, changing their properties and re-organize neural networks plasticity. Indeed, cytokines can induce nociceptive plasticity by the synthesis of local proteins in the peripheral processes of sensory afferents [7,8], and enhance spontaneous post-synaptic currents in the spinal cord by increasing excitatory and suppressing inhibitory synaptic transmissions [7,8]. To date, the depicted vicious cycle is considered as one of the possible mechanisms that plays a role in central sensitization, allowing for the maintenance of pain regardless of disease progression or treatment and leading to its chronification. Furthermore, changes in the higher order circuits result in chronic pain-associated co-morbidities, such as sleep disturbance (e.g., melatonin), mood alteration (e.g., the brain-derived neurotrophic factor), and cognitive decline. These symptoms should be considered when phenotyping chronic pain patients on a molecular and cellular level [8]. Moreover, chronic pain may be one symptom among others (for instance, mood, sleep, cognition, and metabolism), reflecting a multisystem disorder of the brain under cytokine control. The assessment of concentrations in healthy and affected subjects seems to be reasonable, as no normative validated value exists given the range of cytokine concentration [9]. Molecular and cellular profiling may potentially be useful in order to characterize the subsets of chronic pain patients who are more likely and suitable to respond to brain stimulation. The “cytokinic wave” is an early response, which originates from the accessible periphery to flow towards and cross the blood–brain barrier promoting central inflammatory pathways across multiple networks of the brain.

The distinction of brain stimulation responders versus non-responders has been increasingly criticized, leading to recommendations to apply the objective outcome measures (neuroimaging and electrophysiology) of pain-associated brain networks targeting the inter- and intra-individual variability of the underlying brain network, and to consecutively integrate the findings into the selection of the intended brain target and the choice of the applied brain stimulation techniques. In view of the complex and dynamic character of chronic pain disorders and the pathophysiology of the underlying brain circuits, multifocal brain targets (motor cortex and prefrontal cortex) may be considered, in particular, for NIBS. Structural and functional neuroimaging (connectomic neuromodulation), electrophysiological diagnostics (EEG and MEG), digital phenotyping, and molecular inflammatory assays may become useful approaches to counterbalance these inter- and intra-individual variabilities relevant for diagnosis and treatment [10,11].

However, only a few neuromodulation pain studies (such as spinal cord stimulation (SCS), cervical noninvasive vagus nerve stimulation (nVNS), and dorsal root ganglion stimulation) determined the levels of circulating cytokine in the blood, cerebrospinal fluid and saliva for a variety of pain disorders (for instance, primary headache disorders, back pain, neuropathic pain, and complex regional pain syndrome), indicating that the neuromodulation of the spinal cord and peripheral nerve system downregulates pro-inflammatory pathways [9]. 

Nevertheless, neuroinflammatory profiling in chronic pain disorders treated with NIBS or DBS is sparse [9]. Hence, this Editorial calls for increased attention to this area of clinical research studies and emphasizes the potential value of inflammatory assays, not only to objectively quantify brain stimulation responsiveness, but also as a potential and equally useful method for diagnosis and patient selection [10,11]. Hence, the authors advocate the implementation of such inflammatory assays in the treatment algorithms and in the post-procedural period of brain stimulation for chronic pain.

## Data Availability

The data presented in this study are available on request from the corresponding author.

## Abbreviations

NIBS—noninvasive brain stimulation; TMS—transcranial magnetic stimulation; tDCS—transcranial direct current stimulation; tACS—transcranial alternating current stimulation; DBS—deep brain stimulation; MCS-Il—interleukin; TNF—tumor necrosis factor; BDNF—brain derived neurotrophic factor.
